# Factors associated with the receipt of pensions among older adults: ELSI-Brazil

**DOI:** 10.11606/S1518-8787.2018052000665

**Published:** 2018-10-25

**Authors:** Eli Iola Gurgel Andrade, Mariângela Leal Cherchiglia, Paulo Roberto Borges de Souza, Fabíola Bof de Andrade, Juliana Vaz de Melo Mambrini, Maria Fernanda Lima-Costa

**Affiliations:** I Universidade Federal de Minas Gerais. Faculdade de Medicina. Departamento de Medicina Preventiva e Social. Belo Horizonte, MG, Brasil; II Fundação Oswaldo Cruz. Instituto de Informação e Comunicação Científica e Tecnológica. Rio de Janeiro, RJ, Brasil.; III Fundação Oswaldo Cruz. Instituto René Rachou. Núcleo de Estudos em Saúde Pública e Envelhecimento. Belo Horizonte, MG, Brasil; IV Fundação Oswaldo Cruz. Instituto René Rachou. Programa de Pós-Graduação em Saúde Coletiva. Belo Horizonte, MG, Brasil

**Keywords:** Aged, Retirement, Pensions, Health Status, Socioeconomic Factors, Health Surveys, Idoso, Aposentadoria, Pensões, Nível de Saúde, Fatores Socioeconômicos, Inquéritos Epidemiológicos

## Abstract

**OBJECTIVE:**

To describe the prevalence of receipt of pensions and associated factors in a nationally representative sample of the Brazilian population aged 50 years and over.

**METHODS:**

We used data from 9,130 participants from the Brazilian Longitudinal Study of Aging (ELSI-Brazil) baseline survey. The outcome variable was receipt of pensions from any source. The exploratory variables were age, gender, residence by region and by urban/rural area, household arrangements, schooling, household assets, perception of income sufficiency, age when started working, number of chronic diseases, and functional limitation. The analyses were based on the Poisson and binary logistic regressions.

**RESULTS:**

The prevalence of the receipt of pension was 54.3%. In the multivariate analysis, the following factors showed statistically significant (p < 0.05) associations with the outcome: age [Prevalence Ratio (PR) = 2.59 and 3.24 for 60–69 and 70 years], rural residence (PR = 1.23 ), residence in the Northeast, South and Southeast compared to the North (PR ranging from 1.18 to 1.23), living arrangements (PR = 1.07 and 1.15 for living with one person and living alone), perception of income sufficiency (PR = 1.08 and 1.15 for sometimes and always), functional limitation (PR = 1.13) and having 1 and ≥ 2 chronic diseases (PR = 1,09 and 1,17). Negative association was observed for 5-8 years of education. No association between age when the individual started working and the outcome was observed. Younger participants (50–59 years old) with ≥ 2 diseases or functional limitation were 31% and 63% more likely to receive pensions, respectively; the strength of these associations declined with age.

**CONCLUSIONS:**

The results suggest that health conditions are important determinants of early retirement. Discussions to increase age to the retirement cannot be separated from those on improvements in the health conditions of the Brazilian population.

## RESUMO

**OBJETIVO:**

Descrever a prevalência do recebimento de aposentadorias e pensões e analisar seus fatores associados em amostra nacional da população com 50 anos ou mais.

**MÉTODOS:**

Foram utilizados dados de 9.130 participantes da linha de base do Estudo Longitudinal da Saúde dos Idosos Brasileiros (ELSI-Brasil). O desfecho foi o recebimento do benefício por qualquer fonte. As variáveis exploratórias incluíram: idade, sexo, residência por região e zona urbana ou rural, arranjos domiciliares, escolaridade, bens domiciliares, suficiência da renda, idade em que começou a trabalhar, número de doenças crônicas e limitação funcional. As regressões de Poisson e logística binária foram utilizadas nas análises.

**RESULTADOS:**

A prevalência do recebimento do benefício foi de 54,3%. Na análise multivariada, os seguintes fatores apresentaram associações significantes (p < 0,05) com o recebimento do benefício: idade [razão de prevalência (RP) = 2,59 e 3,24 para 60–69 e 70 anos], residência rural (RP = 1,23), residência no Nordeste, Sul e Sudeste em comparação ao Norte (RP variando entre 1,18 e 1,23), arranjos domiciliares (RP = 1,07 e 1,15 para morar com uma pessoa e para morar só), percepção da suficiência da renda (RP = 1,08 e 1,15 para às vezes e sempre suficiente), ter doenças crônicas (RP = 1,09 e 1,17 para 1 e ≥ 2) e limitação funcional (RP = 1,13). Associação negativa foi observada para escolaridade igual a 5–8 anos (RP = 0,88). O recebimento do benefício não foi associado com a idade em que começou a trabalhar. Participantes mais jovens (50–59 anos) com ≥ 2 doenças crônicas ou limitação funcional foram 31% e 63% mais propensos a receber o benefício. Com o aumento da idade, a força dessas associações diminuiu.

**CONCLUSÕES:**

Os resultados sugerem que as condições de saúde são importantes determinantes da aposentadoria ou pensão precoce. As discussões para aumentar a idade da aposentadoria não podem ser separadas daquelas acerca de melhorias das condições de saúde da população brasileira.

## INTRODUCTION

The Brazilian social security system is an important instrument for protection in old age and for the reduction of social inequalities. In 2016, 33.6 million persons received social security benefits under the General Social Security Scheme, that is, more than 80% of the Brazilian population aged 60 and over is currently protected by social security^1^.

The Brazilian social security legislation in force was established in the Federal Constitution of 1988 (CF/1988). In addition to the, which is responsible for the largest population coverage, public workers are covered by the General Social Security Scheme and each State has its own scheme, which cover approximately eight million beneficiaries^2^. Both schemes are public and compulsory, funded by contributions from employees and employers on salaries, taxation on revenue, and net profits of companies and other sources^3^. A third scheme, private and optional, is represented by supplementary pensions, whether open or closed^4^.

Since 1988, several attempts have been made by successive governments to change the Social Security System, either in the rules to access pensions or the systematic removal of public resources to finance public health and social care policies^5^. By the end of 2016, amid an economic package for long-term fiscal adjustment, the government focused mainly on social spending: health and education began to have their budgets frozen for the next twenty years at expense levels equivalent to those of 2017. For social security, the proposal is to increase the minimum age for retirement and other benefits accessible by age, coupled with new requirements that lower the values of pensions and increase the time of contribution for retirement^6^.

The proposal under discussion is based on projections of the effects of population aging on the sustainability of pension schemes. It is estimated that, by 2060, the potential support ratio (i.e., the ratio between the active-age population (15-64 years) and those aged 65 years and over, which is currently approximately 9.0 persons of working age for each older adult] will be reduced to 2.3 over the next 40 years, which is the reality of the main developed countries in Central and Northern Europe^7^.

In countries that have completed their demographic transition, research on aging and social protection has broadened its scope to include, among other issuges, health consequences, such as the provision of long-term care for older adults with functional disabilities, income, work lifecouse, and lifestyles^,^ ^8^ ^-^ ^10^.

In Europe, retirement patterns vary across countries, although their demographic trends are similar. There are three main competing explanations for this: institutional differences (the age limit required for pensions), types of benefits (disability pensions, sickness and unemployment benefits), and differences in health conditions. Furthermore, in Europe, 97% of the persons who can retire have left the labor market; however, age alone is not a determinant of retirement, as it is also associated with other conditions such as type of occupation and educational level^11^. In Latin American countries, the eminently contributory nature of pension schemes favors those with stable and formal work trajectories, associated with gender characteristics and educational opportunities^12^ ^-^ ^14^.

The change in the age criteria for the access to pensions has dominated the current debate about planned reforms of the Brazilian government for social security, which highlights the lack of studies that broaden the scope of the analysis beyond the fiscal and demographic constraints to which it is circumscribed^15^. The objective of this study was to estimate the prevalence and factors associated with the recept of pensions among Brazilian older adults.

## METHODS

### Data Source

The Brazilian Longitudinal Study of Aging (ELSI-Brazil) is a household-based study, whose sample was designed to represent the Brazilian population aged 50 years and over. It is a complex sample that combines different stages of selection, namely, municipalities, census tracts, and households. The inverse sampling process was adopted, with an estimated 10,000 participants (9,412 participated). The research was conducted in 70 municipalities in the five regions of the country. The data collection of the baseline was conducted between 2015 and 2016. The ELSI-Brazil has two questionnaires: household (comprising general information about the household and all residents) and individual (comprising information about each resident aged 50 years and over). For this analysis, data from both sources were used. More details can be found on the research homepage^a^.

### Variables

The dependent variable of this study is the receipt of pension from any source (General Social Security Scheme, Federal, State, or Municipal government, or additional pension plan). The independent variables included the following two domains: (i) sociodemographic characteristics (age, residence in rural or urban area, region of residence, number of persons living in the household, number of complete years of schooling, score of household goods, perceived sufficiency of income for the expenses of the household, and age at which the person started working) and (ii) health conditions (number of chronic diseases and functional disability).

The socioeconomic status of the family was defined by a score of household goods based on principal component analysis and considering the number of household appliances and vehicles, as well as the presence of paid domestic workers. As the score may vary from -∞ to +∞, the analysis considered its distribution in quartiles. The number of chronic diseases was defined by a previous medical diagnosis of hypertension, diabetes, heart disease (infarction, angina, or heart failure), cerebrovascular disease, asthma, chronic obstructive pulmonary disease, arthritis, cancer, or chronic renal failure. Functional disability was defined by the report of any difficulty (little, great, or failure) to perform basic activities of daily living (BADL), i.e., walking across a room, , dressing, bathing, eating, getting in and out of bed, and using the toilet.

### Statistical Analysis

According to the ELSI-Brazil’s sampling desing, results were described in weighted means and prevalences and their 95% confidence intervals (95%CI). For the unadjusted analyses, we used linear regression and t Pearson’s chi-square test , respectively.

The multivariate analysis of factors associated with receipt of pensions was based on prevalence ratios (PR) estimated using Poisson regression. Given the absence of collinearity between the independent variables, the multivariate model was simultaneously adjusted for all of them. Finally, we used binary logistic regression to estimate the predicted probabilities of the receipt of pension, in an analysis stratified by the number of chronic diseases (two or more *versus* one or zero) and functional disability (yes *versus* no). These models were based on the final multivariate analysis, with evaluation of possible multiplicative interactions between age, number of chronic diseases, and functional disability. The results of the predicted probabilities are fitted.

The analyses were performed considering both sexes, since no important differences were observed in the factors associated with the receipt of pensions in the exploratory analysis. All analyses were performed using the procedures for complex samples from the statistical package Stata (College Station, Texas, USA), version 14.2.

### Ethical Aspects

The ELSI-Brazil was approved by the Research Ethics Committee of the *Fundação Oswaldo Cruz*, Minas Gerais (CAAE 34649814.3.0000.5091). All participants signed the informed consent for each of the study procedures.

## RESULTS

Of the 9,412 participants of the baseline of ELSI-Brazil, 9,130 were included in this analysis. Exclusions were due to incomplete information for at least one of the study variables. Among the participants, 54.3% received pension. As we can see in Table 1, the mean age of the participants was 62.9 years, 54.0% were women, 15.2% lived in rural areas, 47.3% lived in the Southeast region, 58.9% lived with two or more persons, 13.3% had never studied, 40.9% reported that income was always insufficient for household expenses, and 60.7% had started working before the age of 15 years. Regarding health conditions, 70.8% had one or more chronic diseases and 15.9% functional disability.

**Table 1:** Sociodemographic characteristics and health conditions of the 9,130 study participants. Brazilian Longitudinal Study of Aging (ELSI-Brazil), 2015–2016.

Characteristic	% or mean^a^	95%CI
Receipt of pension	54.3	51.1–57.4
Age group (years)		
50–59	47.9	43.8–52.08
60–69	29.7	27.9–31.5
70 and over	22.4	19.7–25.4
Mean age (years)	62.9	62.1–63.8
Female	54.0	50.9–57.0
Living in rural area	15.2	11.1–20.5
Region of residence		
North	5.6	2.3–12.8
Northeast	24.2	15.9–34.9
Southeast	47.3	35.6–59.2
South	16.4	8.6–28.9
Midwest	6.6	3.0–13.8
Living arrangements		
Living with two or more persons	58.9	56.3–61.5
Living with one person	32.2	30.1–34.3
Living alone	8.9	8.0–9.9
Complete years of schooling		
Never studied	13.3	10.9–16.0
1–4	38.0	35.8–40.3
5–8	21.6	19.2–24.0
9 or more	27.1	24.8–29.6
Score of household goods (quartiles)		
1st (lowest)	25.0	20.7–29.8
2nd	24.9	23.1–26.7
3rd	25.1	22.8–27.6
4th (highest)	25.1	21.8–28.6
Perception of income sufficiency for household expenses		
Never enough	40.9	37.8–44.1
Sometimes enough	25.7	24.0–27.5
Alwasys enough	33.4	30.9–36.0
Age at which started working (in years)		
5–14	67.0	64.0–69.9
15–25	29.7	27.2–32.2
26 and over	3.3	2.6–4.1
Number of chronic diseases^b^		
None	29.2	27.5–31.0
One	34.8	33.3–36.3
Two or more	36.0	34.1–38.0
Functional disability^c^	15.9	14.6–17.3

^a^ All results are percentages, except when indicated. Estimates were made considering individual weight and sample parameters.

^b^ Medical diagnosis of hypertension, diabetes, heart disease (infarction, angina, or heart failure), cerebrovascular disease, asthma, chronic obstructive pulmonary disease, arthritis, cancer, or chronic renal failure.

^c^ Difficulty in: crossing accross a room, dressing, bathing, eating, getingg in and out of bed, using the toilet.

%: Percentages estimated in relation to the total of the column.

Table 2 shows the distributions of the sociodemographic characteristics and indicators of health conditions according to the receipt of pensions. In the univariate analysis, the following characteristics showed statistically significant associations (p <0.05) with the receipt of pensions: age group, rural or urban residence, living arrangements, schooling, score of household goods, perception of income sufficiency, age at which the person started working, number of chronic diseases, and functional disability.

**Table 2:** Receipt of pension, according to sociodemographic characteristics and health conditions of the 9,130 study participants. Brazilian Longitudinal Study of Aging (ELSI-Brazil), 2015–2016.

Characteristic	%^a^	95%CI	p^d^
Age group (years)			
50–59	26.1	23.7–28.6	< 0.001
60–69	71.1	68.6–73.5	
70 and over	92.3	90.6–93.6	
Sex			
Male	52.2	50.4–58.0	0.952
Female	54.2	50.7–57.9	
Residence			
Urban	52.3	49.1–55.4	< 0.001
Rural	65.4	59.4–71.0	
Region of residence			
North	41.6	33.8–49.8	0.117
Northeast	56.4	50.5–62.1	
Southeast	53.8	48.9–58.6	
South	59.3	50.2–67.8	
Midwest	48.1	38.1–58.4	
Living arrangements			
Living with two or more persons	47.9	44.7–51.2	< 0.001
Living with one person	61.0	57.5–64.4	
Living alone	72.1	67.8–76.0	
Complete years of schooling			
Never studied	71.8	66.7–76.3	< 0.001
1–4	59.3	55.4–63.1	
5–8	42.1	37.9–46.4	
9 or more	48.2	44.1–52.2	
Score of household goods (quartiles)			
1st (lowest)	58.0	53.6–62.3	0.047
2nd	54.4	50.2–58.5	
3rd	53.6	49.2–57.9	
4th (highest)	51.2	47.3–55.1	
Perceived sufficiency of family income for all expenses of the household			
Never enough	47.1	44.2–49.9	< 0.001
Sometimes enough	52.7	48.4–57.0	
Always enough	64.3	60.3–68.2	
Age at which started working (in years)			
5–14	55.7	51.8–59.4	0.021
15–25	50.5	47.2–53.9	
26 and over	54.0	45.2–62.5	
Number of chronic diseases^b^			
None	42.6	38.5–46.8	< 0.001
One	52.9	49.2–56.4	
Two or more	65.1	62.0–68.0	
Functional disability^c^			
No	51.9	48.6–55.3	< 0.001
Yes	66.7	62.9–70.2	

^a^ All results are percentages. Estimates were made considering individual weight and sample parameters.

^b^ Medical diagnosis of hypertension, diabetes, heart disease (infarction, angina, or heart failure), cerebrovascular disease, asthma, chronic obstructive pulmonary disease, arthritis, cancer, or chronic renal failure.

^c^ Difficulty in: crossing across a room, dressing, bathing, eating, getting in and up the bed , using the toilet.

^d^ Pearson’s chi-square test for differences between those who receive the benefit and those who not.

%: Percentages and 95% confidence intervals estimated in relation to the line total.

The statistically significant results of the analysis of the factors associated with the receipt of benefit can be seen in Table 3. Positive and statistically significant associations (p <0.05) were observed for age (PR = 2.59 and 3.24 in the age groups of 60–69 years and 70 years, respectively), residence in the rural area (PR = 1.23), living in the Northeast, Southeast, and South regions, compared to the North region (with PR varying between 1.18 and 1.23), living arrangements (PR = 1.07 for living with one person and PR = 1.15 for living alone), perception of income sufficiency (PR = 1.08 for sometimes and PR = 1.15 for always), having one or more chronic diseases (PR = 1.09 and 1.17, respectively), and having functional disability (PR = 1.13). We observed a negative and statistically significant association for schooling equal to 5–8 years (PR = 0.88 compared to those who never studied).

**Table 3:** Statistically significant results of the multivariate analysis of the association between sociodemographic characteristics and health conditions and the receipt of pension among the 9,130 study participants. Brazilian Longitudinal Study of Aging (ELSI-Brazil), 2015–2016.

Characteristic	Prevalence ratio^a^	95%CI^a^
Age group (*versus* 50–59 years)		
60–69	2.59	2.37–2.83
70 and over	3.24	2.95–3.55
Rural residence (*versus* urban)	1.23	1.13–1.34
Region of residence (*versus* North)		
Northeast	1.21	1.08–1.35
Southeast	1.18	1.06–1.32
South	1.23	1.07–1.41
Midwest	1.10	0.95–1.27
Living arrangements (*versus* living with two or more persons)		
Living with one person	1.07	1.03–1.12
Living alone	1.15	1.09–1.22
Complete years of schooling (*versus* never studied)		
1–4	0.96	0.92–1.00
5–8	0.88	0.81–0.96
9 or more	1.02	0.93–1.11
Perception of income sufficiency for household expenses (*vs*. never enough)		
Sometimes enough	1.08	1.03–1.14
Always enough	1.15	1.09–1.21
Number of chronic diseases^b^		
One	1.09	1.03–1.15
Two or more	1.17	1.10–1.24
Functional disability (*versus* no)^c^	1.13	1.07–1.20

^a^ Prevalence ratio and 95% confidence interval estimated by robust Poisson regression, considering individual weights and sample parameters, and adjusted for all variables listed in the table plus sex, score of household goods (in quartiles), and age at which the person started working.

^b^ Medical diagnosis of hypertension, diabetes, heart disease (infarction, angina, or heart failure), cerebrovascular disease, asthma, chronic obstructive pulmonary disease, arthritis, cancer, or chronic renal failure.

^c^ Difficulty in: crossing across a room, dressing, bathing, eating, getting in and out the bed, using the toilet.

**Figure 1:**
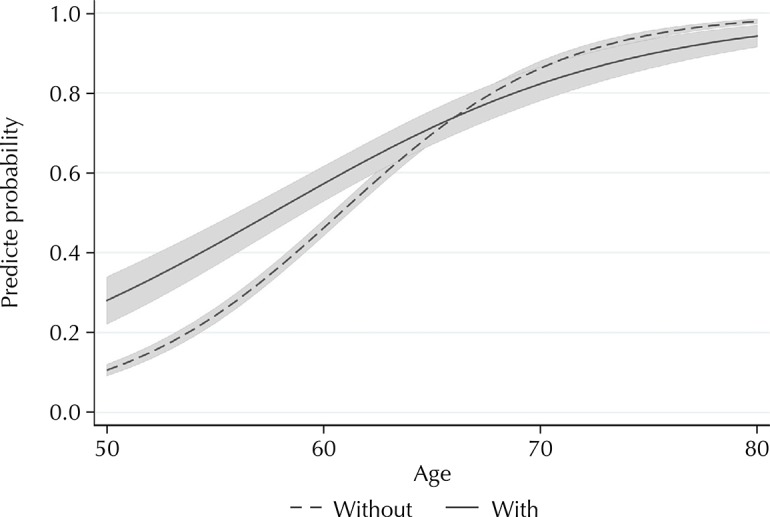
Predicted probabilitiesa and r 95% confidence intervalsb of the receipt of pension among the 9,130 study participants by age, according to the number of chronic diseasesc. Brazilian Longitudinal Study of Aging (ELSI-Brazil), 2015–2016.

^a^ Probabilities estimated by binomial logistic regression, considering individual weights and sample parameters, and adjusted for age, rural/urban residence, living arrangements, schooling, perceived income sufficiency, functional disability, sex, score of household goods (in quartiles), and age at which the individual started working.

^b^ Shaded area.

^c^ Medical diagnosis of hypertension, diabetes, heart disease (infarction, angina, or heart failure), cerebrovascular disease, asthma, chronic obstructive pulmonary disease, arthritis, cancer, or chronic renal failure.

**Figure 2:**
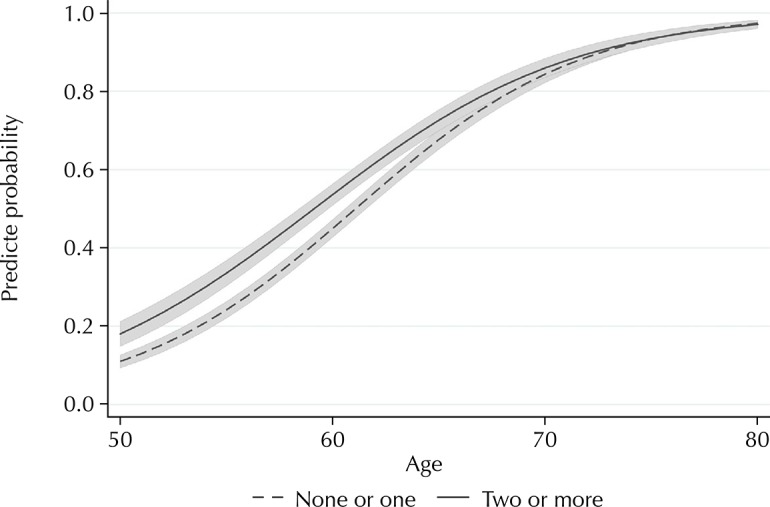
Predicted probabilitiesa and 95% confidence intervalsb of the receipt of pension among the 9,130 study participants by age, according to functional disabilityc. Brazilian Longitudinal Study of Aging (ELSI-Brazil), 2015–2016.

^a^ Probabilities estimated by binomial logistic regression, considering individual weights and sample parameters, and adjusted for age, rural/urban residence, living arrangements, schooling, perceived income sufficiency, number of chronic diseases, sex, score of household goods (in quartiles), and age at which the individual started working.

^b^ Shaded area.

^c^ Difficulty in: crossing across a room, , dressing, bathing, eating, getting in and up the bed, using the toilet.

Figures 1 and 2 show the predicted probabilities of the receipt of pension by age, according to number of chronic diseases (two or more *versus* one or none) and functional disability (yes *versus* no), respectively. The probabilities of the receipt of pension among those with two or more diseases and those with functional disability were clearly higher at younger ages compared to older ages (p value for interaction < 0.001 in each of these models).

## DISCUSSION

The results of this study show that age was the strongest factor associated with the receipt of pension, as expected. Other associated factors were: living in a rural area, living alone or with one person, perception that income is always or sometimes enough for household expenses, and having chronic diseases and functional disability. Residents in the North and Northeast regions were less likely to receive the benefit compared to those in other regions. The receipt of pensions did not show an association with sex, age at which the person started working, and score of household goods.

The Organization for Economic Cooperation and Development (OECD)^16^ considers that the coverage of workers through one or more pension schemes is essential to avoid poverty at older ages. The results of this study show that the proportion of the receipt of pensions among Brazilians is 26% at 50–59 years, 71% at 60–69 years, and it reaches almost universal coverage (92%) at 70 years and over. This coverage is much higher than that observed for older populations in Latin America and the Caribbean (approximately 40%)^13^.

The high social security coverage in Brazil represents an important advance, considering that men and women had a late access to labor relations protected by social security law in the country ^17^. In 1923, with Law Elói Chaves, urban workers assumed the leading role in the institution of retirement rights. However, a new constitutional milestone was reached only after 60 years in 1988, when the rights to social protection were universalized for urban and rural workers, older adults, and disabled persons ^13^. Men and women in the countryside, whether married or not, working as employees or in the family economy, became entitled to retire at 60 and 55 years, respectively, whose benefit cannot be less than one minimum wage. The previous scheme established the limit equivalent to half a minimum wage for rural pensions, and pensions were limited to 30% of the main benefit^18^.

We need to recognize the impact of the universalization of social security on the living conditions of the rural population and families in Brazil. The extension of pensions to women under any condition (partner or spouse) was responsible for one of the most marked advances in the agenda of sex equity among rural workers^19^.

Our results show a greater coverage of pensions in rural areas than in urban areas. Valadares and Galiza^20^ state that 99% of the rural pensions in Brazil were by age in 2015. For the authors, social security is now the main source of income for rural families, which ensures the movement of service and trade sectors in most Brazilian municipalities. In this scenario, the authors emphasize that the rural pension system functions as one of the distributive pillars of social security in Brazil, together with the right to health and care.

However, we need to note the increasing difficulty of the older population to access the benefit of special retirement. In fact, while proof of registration in the National Register of Social Information (CNIS) is enough for urban workers for the recognition of relations and contributions, for special insured individuals, the lack of a public and unified registry service weakens them and exposes them to all the diversity of local administrative demands. In recent years, according to Valadares and Galiza^20^, approximately 30% of rural pensions were denied by the administration but ensured by Justice. These difficulties may explain, in part, the heterogeneity in the granting of pensions by regions, with a lower proportion of beneficiaries in the North and Midwest regions, as observed in this analysis.

The type of social security coverage in Brazil gives great importance to the duration and quality of employment relations throughout the workers’ lives^21^. For this reason, it is evident the difficulty of the worker, submitted to a flexible and heterogeneous labor market, to meet the requirement of high and continuous monthly contributions to social security. The combination of a high unemployment rate, low levels of pay, turnover, and high levels of informality means that two-thirds of pensions are granted by age, with the minimum benefit.

The results of this study are in line with such observations. Although 67% of the participants declared beginning their work life between the ages of five and 14, the non-association between the receipt of pension and the age of entry into the labor market reveals the preponderance of the age limit as the main attribute of access to the benefit^22^. We also observed that never having studied and having more than nine years of study are associated with the receipt of benefits. The possible explanation may lie in the characteristics of access to pensions. On the one hand, we can find rural workers and beneficiaries by age, both populations with lower schooling^23^. On the other hand, we can mention workers with an intermediate and higher education level who have a with higher probability of regular occupational trajectories, which is a fundamental criterion for the qualification of the benefit^20^.

Living arrangements in old age vary between countries. In high income countries, older adults tend to live independently (alone or with a partner), while in middle-low income countries t is more common to live with children or other relatives. Among Latin American countries, the percentage of older persons living alone or with their spouse is higher in Uruguay and Argentina and lower in Honduras and Nicaragua^24^. In our analysis, the proportion of older adults living with two or more persons predominated; however, our analysis show a positive association between the receipt of pension and living alone or with one person. These results suggest that the receipt of pensions favors the option of living independently.

Another positive aspect to be highlighted is the perception of income sufficiency. The results point out to the greater economic security of those who receive pensions. The perception that income was always or sometimes enough to cover household expenses was significantly more frequent among those receiving the benefit.

Aged populations have a higher prevalence of chronic diseases and disabilities, which generates challenges for the social and health systems^25^. To understand the relation between the receipt of pension and presence of one or more chronic diseases or functional disability, we need to consider the heterogeneity of the segment of older adults. This group has had varied experiences and faces different life conditions in old age because of social, sex, and racial inequalities, access to and quality of health services, occupational and environmental factors, and health behaviors^26^. The guidelines from the national policy of older adults recognize the centrality of the loss of functional ability (physical and mental abilities to perform basic and instrumental activities of daily living) in the quality of life of older adults^27^ ^,^ ^28^.

In this study, 71% of the participants had one or more chronic diseases and 16% had limitations to perform basic activities of daily living. This proportion occupies an intermediate position between that observed in Spain, England, and the United States^29^. Our results showed an independent association between having one or more chronic diseases and receiving pensions. Similar association was observed for functional disability. Given the cross-sectional nature of this analysis, it is not possible to know whether the worst health conditions preceded or were subsequent to the receipt of pensions. However, longitudinal studies conducted in Canada, the United States, and Europe show that these conditions are determinants for the decision to retire^30^.

The predicted probabilities of the receipt of the benefit among those with two or more chronic diseases, as well as those with functional disability, were higher at younger ages compared to higher age groups. These results show that health conditions are associated with the early receipt of the benefit. We found an important age difference: younger persons (50–59 years old) with two or more chronic diseases were 31% more likely to receive the benefit. This value was 63% for functional disability. With increasing age, the strength of these associations gradually decreased. These results suggest that health conditions are important determinants of early retirement.

This study has advantages and limitations. Among the advantages, we can mention its large population-based sample. Among the limitations, we can mention the cross-sectional nature of the study, which does not allow establishing temporal relations between the independent variables and the receipt of pension. Additionally, the ELSI-Brazil questionnaire may have contributed to reduce the strength of the associations observed by not allowing separation between the source and type of benefit. Finally, the self-reported definition of the receipt of benefit is subject to memory or information bias, but it is important to note that the prevalence of the receipt of benefit observed in this analysis was similar to those recorded in the Brazilian Statistical Yearbook of Social Security ^1^.

The results of this analysis, based on a representative sample of the Brazilian population aged 50 years and over, show that the receipt of pensions is associated with the perception of greater economic security and autonomy, which is determined by the option of living independently. Finally, the evidence points to the fact that discussions to raise the retirement age should not be separated from those on improving the health conditions of the Brazilian population, since our results show that the early receipt of the benefit is associated with worse health conditions and functional disability.

## Footnotes

a Fundação Oswaldo Cruz. Brazilian Longitudinal Study of Aging. Rio de Janeiro; c2015 [cited 2017 Nov 28]. Available from: http://elsi.cpqrr.fiocruz.br
